# Increased Serum Neuropeptide Galanin Level Is a Predictor of Cognitive Dysfunction in Patients with Hip Fracture

**DOI:** 10.1155/2021/9141978

**Published:** 2021-12-10

**Authors:** Zichao Xue, Ke Zhang, Biao Luo, Long Fan, Ruizhe Zhao, Guangliang Hu

**Affiliations:** ^1^Department of Orthopaedic Surgery, Qingdao Municipal Hospital, Qingdao, China; ^2^Department of Anesthesia Operating, Qingdao Municipal Hospital, Qingdao, China

## Abstract

**Background:**

Hip fracture is a common occurrence in elderly populations and is frequently followed by various levels of cognitive dysfunction, leading to adverse functional outcomes. Risk stratification of hip fracture patients to identify high-risk subsets can enable improved strategies to mitigate cognitive complications. The neuropeptide galanin has multiple neurological functions, and altered levels are documented in dementia-type and depression disorders. The present study investigated the association of serum neuropeptide galanin levels in hip fracture patients with the occurrence of cognitive dysfunction during the first week of admission.

**Methods:**

276 hip fracture patients without preexisting delirium, cognitive impairment, or severe mental disorders were included in a cross-sectional study. Serum galanin levels were assessed by ELISA on the second day of admission. Routine clinical and laboratory variables were documented. MoCA was performed within 1 week, and those with a score < 26 were categorized with “cognitive decline.” Inferential statistics including multiple linear regression analysis were applied to determine the association of serum galanin level and cognitive status.

**Results:**

141 patients were categorized with “cognitive decline,” and 135 patients were categorized as “cognitively normal.” Serum galanin was highly significantly increased in the “cognitive decline” group (34.2 ± 4.8, pg/ml) compared to the “cognitively normal” group (28.9 ± 3.7, pg/ml) and showed significant negative correlation with MoCA scores (*r* = −0.229, *p* = 0.016). Regression analysis showed serum galanin as the sole significant independent predictor of lower MoCA scores (*β* = 0.231, *p* = 0.035) while age, gender, blood pressure, cholesterol, and blood glucose levels had no significant association.

**Conclusion:**

Higher serum galanin predicted the development of cognitive dysfunction and worse MoCA scores in a cohort of hip fracture patients without preexisting cognitive impairment or delirium at admission, thus warranting large-scale studies investigating galanin as a candidate biomarker to identify hip fracture patients at risk of cognitive decline.

## 1. Introduction

Hip fracture is a commonly occurring cause for morbidity and mortality among older individuals [1–3]. Ageing populations are anticipated to account for further increase in hip fracture incidence in the coming decades, leading to significant social and healthcare burden globally [4]. Hip fractures are fraught with a number of medical and surgical secondary complications in the perioperative period [5]. Among these, cognitive complications are reported to occur at a frequency of at least 10%, particularly affecting elderly patients [6], with postoperative delirium affecting up to 30% of patients [7]. A multifactorial causal and risk factor profile has been associated with acute postoperative cognitive dysfunction after hip fracture, including higher age, preexisting medical or cognitive impairment, preoperative medications, anesthetic agents, infections, and electrolyte disturbances [8, 9]. Postoperative delirium, along with other factors such as history of stroke and perioperative blood loss, imposes risk for subsequent cognitive decline after hip fracture [10–12], which can substantially worsen patients' quality of life and cause functional impairment. Postoperative cognitive dysfunction or decline in the first week after hip fracture has been documented at a rate of 32% and linked to worse outcomes [13], highlighting a need to identify subsets of patients at high-risk for tailored preventive or mitigation efforts.

The identification of predictive biomarkers that can enable early risk stratification of hip fracture patients susceptible to cognitive complications can thus have high clinical relevance. The highly inducible neuropeptide galanin is a neuromodulator ubiquitously present in the central and peripheral nervous systems as well as nonneural tissues, with 3 known G-protein coupled receptors, GalR1, GalR2 and GalR3, and galaninergic signaling has been implicated a range of functions including memory and attention processing, mood, feeding behavior, pain, and tumor pathogenesis [14, 15]. Alzheimer's and Lewy body type dementias are marked by progressive overexpression of galanin in the brain in the cerebral cortex, hippocampus, and basal forebrain [16, 17]. Galanin can regulate basal forebrain cholinergic neurons that are the main mode of cholinergic transmission in the cortex and hippocampus [14, 15]. Basal forebrain cholinergic neurons that degenerate early in course of Alzheimer's disease are marked by heightened galanin expression, which supports the notion that galanin may play a role in cognitive dysfunction development. In support, the central administration of galanin was found to cause deficits in a variety of memory and learning tasks in rodents [15, 16, 18]. One of the mechanisms of galanin-induced impairment in cognition is its down regulation of cholinergic transmission, supported by the finding that beneficial effects of acetylcholine administration to rodents with cholinergic lesions in selected brain regions were blocked by administration of galanin [19]; thus, galanin appeared to exacerbate cognitive impairment and limit long term potentiation [20] arising from cholinergic deficit in brain disorders. However, others have noted neuroprotective effects of galanin in the hippocampus [21] and basal forebrain cholinergic neurons [22]. These findings, together with data showing that galanin overexpression upregulates genes promoting the survival of cholinergic neurons [14, 23], have led to the emergence of the current notion that the galanin overexpression is induced in response to neuronal injury as a mechanism supporting the survival of cholinergic neurons in late stages of dementia.

While the functional implications of galanin plasticity in the brain during cognitive decline seem to be complex and tightly regulated [23], and are yet to be fully elucidated, the manipulation of galanin presents a significant potential opportunity for slowing cognitive disorders. The role of galanin in mood and cognitive function has also highlighted its potential value as a biomarker and molecular therapeutic target [18]. As such, blood galanin levels have been documented as a biomarker for depression [24] and autism spectrum disorders [25]. In an animal model of orthopedic fracture, early postoperative up regulation of CNS galanin levels has been documented [26], where 3 days after tibial fracture surgery in rats, dorsal root ganglion neurons showed a transient upregulation of galanin expression, which declined in the following 2 weeks, and the authors concluded such increase might be correlated to the extent of the neuronal injury. Functionally, galanin is elevated in neuronal injury and closely linked to differentially modulating pain sensation via its receptor-specific actions [27] and may thus act as a bidirectional link between postsurgical pain, neuroimmune system activation, and neurological dysfunction. Surgical injury, neuronal pain, and cognitive dysfunction are interrelated in complex and yet to be fully understood mechanisms, and the current understanding is that surgical injury precipitates a state of neuroinflammation via multiple mechanisms including blood-brain barrier disruption [28] and microglial activation [29], leading to transient or irreversible neurological dysfunction manifesting as cognitive decline or dementia in vulnerable subjects, particularly the elderly [28–30]. Galanin is a participant in this cascade, possibly via multiple mechanisms such as serving as chemoattractant for microglial cells and participating in their activation via phospholipase C and protein kinase C pathways [31]. Overall, the literature supports a hypothesis that circulating galanin could be altered after fracture and correlate with neurological dysfunction owing to neuroimmune activation. The present observational study is thus aimed at assessing serum galanin level as a predictive biomarker of cognitive dysfunction among patients with hip fracture.

## 2. Methods

### 2.1. Study Population and Data Collection

This study protocol was approved by the institutional ethics committee of Qingdao Municipal hospital, and all study procedures were compliant with the Declaration of Helsinki. All patients or legal guardians provided signed written informed consent before participation in the study. Eligible patients with hip fractures were recruited, which included a total of 276 patients with confirmed hip fractures. The inclusion criteria were as follows: adult patients with hip fracture within 1 week of occurrence and willingness for inclusion in the study. The exclusion criteria were as follows: previous history of cognitive impairment, patients diagnosed with delirium by the confusion assessment method [32], previous history of severe mental illness, drug or alcohol dependence, and patients who could not cooperate with the examination procedures.

The patient's age, gender, and previous medical history were determined through interviews, medical record reviews, and a complete physical examination. Blood pressure was measured by an electronic sphygmomanometer. Routine laboratory parameters including low-density lipoprotein (LDL), high-density lipoprotein (HDL), and fasting blood glucose (FBG) were tested by standard laboratory methods at the hospital laboratory.

### 2.2. Cognitive Function Evaluation

MoCA score, a well-established cognitive assessment tool, was used to assess the patients' cognitive status within 1 week of admission as reported earlier [33]. The criterion for cognitive impairment was a MoCA score less than 26 points.

### 2.3. Determination of Serum Neuropeptide Galanin Level

On the second day after admission, the serum galanin level was measured after overnight fasting. A sandwich enzyme-linked immunosorbent assay (ELISA) was performed using commercially available reagents (Abcam, Cambridge, UK) based on the manufacturer's instructions.

### 2.4. Statistical Analysis

Based on the MoCA cut-off score, the patients were grouped into “cognitive decline” or “cognitively normal” groups. Clinical and laboratory variables were compared between the groups using the *t*-test for continuous variables or the chi-square test for dichotomous variables. Pearson's correlation analysis was used to evaluate the correlation between MoCA scores, galanin level, and other baseline variables. Multiple linear regression analysis was performed for MoCA score as the independent variable, using serum neuropeptide galanin level and other baseline variables as predictors to account for the potential impact of various documented risk factors. All statistical analyses were performed using SPSS software version 22.0. A *p* value less than 0.05 was considered statistically significant.

## 3. Results

### 3.1. Study Population

A total of 276 hip fracture patients were eventually included in the study. The clinical and laboratory data for these patients are summarized in Table 1. The mean age of patients was 71.6 ± 9.3 years, of which 188 (68.1%) were male. The mean serum neuropeptide galanin level and MoCA score were (31.6 ± 4.3) pg/ml and (26.0 ± 1.2) points, respectively.

**Figure 1:**
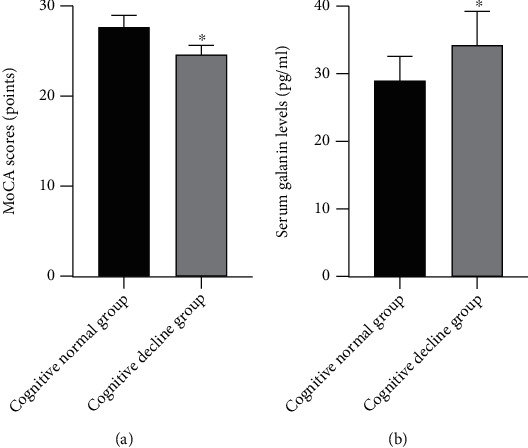
Comparison of MoCA scores and serum galanin levels between the two groups. ^∗^*p* < 0.001.

Based on the MoCA score, 141 patients were categorized into the “cognitive decline” group and 135 patients into the “cognitively normal” group. The clinical and laboratory variables for the two groups are summarized in Table 2. No significant differences between the two groups of patients were noted for age, gender, LDL, HDL, FBG, SBP, and DBP (*p* > 0.05). Mean serum neuropeptide galanin level of the “cognitive decline” group was significantly higher as compared to that of the “cognitive decline” group (Figure 1).

### 3.2. Correlation Analysis

The correlation analysis between the MoCA scores with clinical and laboratory variables showed a significant negative correlation of MoCA score with the serum neuropeptide galanin level (*r* = −0.229, *p* = 0.016) (Table 3).

### 3.3. Regression Analysis

Linear regression analysis showed serum neuropeptide galanin level as a significant independent predictor of cognitive impairment in patients with hip fracture (*β* = 0.231, *p* = 0.035). The other variables in the model included age, gender, blood pressure, LDL, HDL, and FBG and were not significantly associated with MoCA score (*p* > 0.05) (Table 4).

## 4. Discussion

In the present cross-sectional observational study, early serum neuropeptide galanin level measured on the second day of admission was identified as a significant predictor of cognitive decline in the first week of hospitalization in a cohort of patients. As this finding pertained to patients without preexisting cognitive impairment, severe mental illness or those diagnosed with delirium, which are known predisposing factors for cognitive decline [7–9], serum neuropeptide galanin levels may be a potentially valuable biomarker for improved prediction of cognitive decline after hip fracture. The groups with and without cognitive decline did not significantly differ in terms of age, gender, blood pressure, lipid, and glucose parameters, and no significant correlations of background variables with MoCA scores were noted. Postoperative delirium and cognitive dysfunction are the two main cognitive complications in elderly patients [34]. Earlier studies have found advancing age and blood glucose levels significantly increased the odds of postoperative delirium and cognitive dysfunction [35, 36]; however, risk prediction models for postoperative cognitive decline based on clinical and routine laboratory variables lack precision [12, 13] as reflected in current cohort where both groups had comparable baseline profiles. In a related finding, a previous study reported that 65% of hip fracture patients who developed acute postoperative cognitive dysfunction did not have any medical comorbidity [13], underscoring the need to identify hitherto unknown risk markers. Galanin concentration in the peripheral circulation is reported to be a biomarker of severe depression [24], and polymorphisms in the galanin-associated genes are linked to mood disorder susceptibility [37] suggesting that individual differences that may similarly exist in neuropeptide responses to injurious stimuli could contribute to differences in susceptibility for neurological sequelae after major fracture or surgery. The present data indicated that serum galanin levels similarly predicted worse MoCA scores in hip fracture patients after controlling for several potential confounders. Galanin is a pleiotropic neuropeptide modulating learning, memory, and stress response [14–17], and its increased neuronal expression has been documented immediately following orthopedic surgery [26], which may be reflected by early postoccurrence circulating galanin levels as assessed in the present study. Overall, the current results demonstrate that the posthip fracture cognitive dysfunction was associated with a disturbance of the galaninergic signaling system, and therefore, serum galanin is a plausible candidate biomarker for identifying hip fracture patients at high risk for cognitive complications.

The present investigation has several limitations. Single time-point MoCA scores were obtained during the first week after fracture; therefore, any temporal relationship between change in cognitive function and change in galanin levels could not be deciphered. Furthermore, potential variability in the baseline MoCA scores among individuals was not accounted for; although, patients presenting with diagnosed delirium or known severe mental illness were excluded. Data regarding other potential risk variables such as type of anesthesia, serum albumin levels, electrolyte levels, pain [8, 35, 36], severity of the fracture, and dynamic changes of galanin levels were not analyzed. The present study did not identify a clinically relevant value for galanin that predicted cognitive impairment. In future large-scale studies, cut-off levels of serum galanin that predict significant cognitive decline after hip fracture may be identified by receiver operating curve analysis and validated in prospective trials. Such data could enable point of care testing kits that enable high precision prognostic profiling of patients. The presented data also suggest a potential for galaninergic signaling manipulation via galanin receptor ligands [38] to prevent cognitive complications after hip fracture; although, the research in this domain is in its infancy [39, 40].

## 5. Conclusions

Serum neuropeptide galanin levels were significantly higher in hip fracture patients who developed cognitive dysfunction in the first week after admission than in those who did demonstrate cognitive dysfunction. Higher serum galanin levels predicted lower MoCA scores after adjusting for age, gender, blood pressure, glucose, and lipid levels. Early serum neuropeptide galanin level emerged as a candidate biomarker for risk stratification of hip fracture patients for subsequent cognitive dysfunction.

## Figures and Tables

**Table 1:** Descriptive characteristics of hip fracture patients.

Characteristics	Outcomes
Age, years	71.6 ± 9.3
Gender, male, *n* (%)	188 (68.1)
LDL, mmol/L	2.7 ± 0.9
HDL, mmol/L	1.3 ± 0.3
FBG, mmol/L	7.0 ± 0.5
SBP, mmHg	142.2 ± 11.0
DBP, mmHg	90.8 ± 8.9
MoCA, scores	26.0 ± 1.2
Galanin, pg/ml	31.6 ± 4.3

LDL: low-density lipoprotein; HDL: high-density lipoprotein; FBG: fasting blood glucose; SBP: systolic pressure; DBP: diastolic pressure; MoCA: Montreal Cognitive Assessment.

**Table 2:** Clinical characteristics of hip fracture patients based on MoCA.

Characteristics	Cognitive normal group (*n* = 135)	Cognitive decline group (*n* = 141)	*p*
Age, years	71.3 ± 8.9	71.8 ± 9.6	0.654
Gender, male, *n* (%)	90 (66.7)	98 (69.5)	0.613
LDL, mmol/L	2.7 ± 1.0	2.6 ± 0.9	0.383
HDL, mmol/L	1.3 ± 0.2	1.3 ± 0.3	1.000
FBG, mmol/L	6.9 ± 0.6	7.0 ± 0.5	0.133
SBP, mmHg	141.8 ± 10.3	142.6 ± 11.7	0.548
DBP, mmHg	90.5 ± 8.6	91.1 ± 9.2	0.577
MoCA, scores	27.6 ± 1.3	24.5 ± 1.1	<0.001^∗^
Galanin, pg/ml	28.9 ± 3.7	34.2 ± 4.8	<0.001^∗^

LDL: low-density lipoprotein; HDL: high-density lipoprotein; FBG: fasting blood glucose; SBP: systolic pressure; DBP: diastolic pressure; MoCA: Montreal Cognitive Assessment. ^∗^*p* < 0.001.

**Table 3:** Correlation of MoCA score with clinical and laboratory variables.

Characteristics	*r*	*p*
Age	-0.182	0.337
LDL	0.213	0.472
HDL	0.235	0.364
FBG	0.261	0.103
SBP	0.156	0.348
DBP	0.207	0.295
Galanin	-0.229	0.016^∗^

LDL: low-density lipoprotein; HDL: high-density lipoprotein; FBG: fasting blood glucose; SBP: systolic pressure; DBP: diastolic pressure; MoCA: Montreal Cognitive Assessment. ^∗^*p* < 0.05.

**Table 4:** Influence of serum galanin levels on MoCA scores.

Characteristics	*β*	*p*
Age	0.209	0.166
Gender	0.147	0.098
LDL	0.138	0.232
HDL	0.154	0.243
FBG	0.142	0.129
SBP	0.243	0.154
DBP	0.076	0.307
Galanin	0.231	0.035∗

LDL: low-density lipoprotein; HDL: high-density lipoprotein; FBG: fasting blood glucose; SBP: systolic pressure; DBP: diastolic pressure; MoCA: Montreal Cognitive Assessment. ^∗^*p* < 0.05.

## Data Availability

The data presented in the study may be made available from the corresponding author upon reasonable request.
